# Plaies abdominales par arme à feu: expériences des urgences CHU Hassan II, Fès, Maroc

**DOI:** 10.11604/pamj.2018.30.265.9133

**Published:** 2018-08-08

**Authors:** Ahmed Zerhouni, Imane Toughrai

**Affiliations:** 1 Service de CHB, CHU Hassan II, Fès, Maroc; 2 Service de Chirurgie Digestive et Endocrinienne, CHU Hassan II, Fès, Maroc

**Keywords:** Arme a feu, projectiles, plaies de l´abdomen, Firearm, shots, abdominal wounds

## Abstract

We report the case of a young couple, a 30-year old man and his 21-year old wife victim, after a family conflict, of shotgun shooting. Shotgun is allowed in Morocco during the game hunting season. Surgical exploration of both patients showed multiple lesions of the digestive tract as well vascular wound on the man’s femoral artery causing hemorrhagic shock. The study of gunshot wounds is based on resulting tissues lesions which vary as a function of the different shooting parameters. The impact, the path and the found projectile allow detect the type of weapons and the shooting conditions. Gunshot injuries may be very different. They depend on: the weapon in question, the trajectory of the projectile in relation to the hit body, the shooting distance and, especially, the characteristics of the projectile. Contused wounds are most common, with very specific features. They are caused by a criminal act, a suicidal act or an accidental act.

## Image en médecine

Jeune couple, un homme de 30 ans et sa femme de 21 ans victimes d’une fusillade suite à un conflit familiale, par un fusil de chasse autorisé au Maroc lors de la saison de chasse aux gibiers. A leur arrivée au centre hospitalier, l’homme est inconscient, tachycarde à 120 b/min avec une tension artérielle a 70/30 mmHg. A l’inspection de son abdomen on a observé la présence de plusieurs plaies pénétrantes au niveau du pubis du Scarpa et la face antérieure de la cuisse droite, un hématome en regard et abolition du pouls fémorale homolatérale, après la mise en condition, l’exploration chirurgicale a montré une plaie de l’artère fémorale droite. L’hémostase est obtenue après la résection de la partie lésée. Une exploration de la cavité abdominale a révélé la présence de plusieurs plaies grêliques. Le geste a consisté à une résection de la partie perforée avec confection d’une anastomose iléo-iléale. Pour la jeune dame, à son admission elle était stable sur le plan hémodynamique, la présence de plusieurs plaies au niveau de l’abdomen. Vu l’état stable de la patiente on a complété par une tomodensitométrie qui a objectivé la présence de plusieurs projectiles pénétrant le tube digestif avec présence d’un pneumopéritoine. L’exploration chirurgicale a objectivé la présence d’une partie jéjunale crible de projectile ainsi le colon transverse le geste a consisté à une résection du grêle perforé ainsi la résection du colon transverse avec confection d’une anastomose grelo-grelique et une autre anastomose colo-colique.

**Figure 1:**
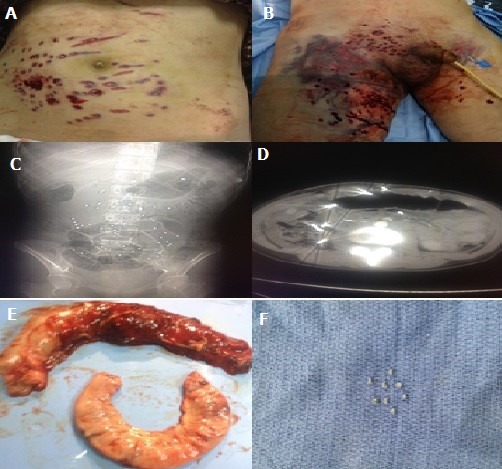
(A,B) images montrant les plaies pariétale par l’arme a feu; (C,D) 2 images scannographiques montrant les projectiles en intra-péritonéale; (E,F) pièce opératoire de résection grelique et colique

